# Supplementary material for: Content-rich biological network constructed by mining PubMed abstracts
Source: BMC Bioinformatics. 2004 Oct 8;5:147. doi: 10.1186/1471-2105-5-147 (PMC528731; doi:10.1186/1471-2105-5-147)
Supplement: Additional File 2 — The original results of the above study (non-essential files are deleted to keep the file size under the limit set by BMC bioinformatics). [file 1471-2105-5-147-S2.bz2 › chilibotAdditionalFile2/dip05/443ID9169459E10419/html/CCL20_CCR6.html]

 


 **CCL20** and **CCR6** 
  
Found 51 abstracts in PubMed, retrieved 05.  
 

 What does Google say? 
 PDF only 
| .edu only 

---

**Interactive relationship** (e.g. stimulation, inhibition, etc)

**Neutral relationship**- MIP 3alpha  **CCL20**  is the unique chemokine ligand for  **CCR6** , a receptor with a restricted distribution.  Ref: 12760962 Am J Respir Cell Mol Biol< MedlineTA>Am J Respir Cell Mol Biol,
- Only on follicular B cells the cognate receptor for  **CCL20** , CC chemokine receptor 6  [ **CCR6** ]   **CCR6** , was expressed strongly and a functional transmigration assay with  **CCR6**  negative B cells demonstrated conclusively the sole signaling of  **CCL20**  through  **CCR6** .  Ref: 12816871 Blood< MedlineTA>Blood,
- Preincubation with the  **CCR6**  ligand  **CCL20**  induced liver sorted tumour cells to preferentially colonize the liver, demonstrating an association between liver metastasis and  **CCR6**  expression in the mouse.  Ref: 12791091 Scand J Immunol, 2003
- Chemokine ligand 20  [ **CCL20** ]   **CCL20**  and human beta defensins HBDs share structural and functional properties, including anti parallel beta pleated sheet core structures, charge distribution, and signaling to adaptive immune cells via the highly selective  **CCR6**  receptor.  Ref: 12748059 Am J Respir Cell Mol Biol< MedlineTA>Am J Respir Cell Mol Biol, 2003

**Non-interactive relationship** (e.g. studied together, co-existance, homology, etc.)

- Because human liver constitutively expresses  **CCL20** , it could attract and select  **CCR6**  cancer cells.  Ref: 12791091 Scand J Immunol, 2003
- Desensitization of  **CCR6**  on naive B cells with  **CCL20**  resulted in receptor downregulation and reduced B cell adhesion.  Ref: 12816871 Blood< MedlineTA>Blood, 2003
- MIP 3alpha  **CCL20**  induces selective migration of DCs because  **CCR6**  is expressed on some immature DCs but not on CD14 DC precursors or mature DCs.  Ref: 12760962 Am J Respir Cell Mol Biol< MedlineTA>Am J Respir Cell Mol Biol, 2003
